# Influence of High-Intensity Ultrasound on Characteristics and Bioaccessibility of Pea Protein in Fiber-Enriched Suspensions

**DOI:** 10.3390/foods12173160

**Published:** 2023-08-23

**Authors:** Ann-Marie Kalla-Bertholdt, Anne Kathrin Baier, Cornelia Rauh

**Affiliations:** Department of Food Biotechnology and Food Process Engineering, Technische Universität Berlin, Koenigin-Luise-Str. 22, 14195 Berlin, Germany

**Keywords:** sonication, pea protein, dietary fiber, bioaccessibility, in vitro digestion

## Abstract

Pea protein is of high interest for the food industry owing to its low allergenicity and high nutritional value. However, it often exhibits poor functionality, such as low solubility. The presence of dietary fiber in food products is beneficial for human health but may decrease the bioaccessibility of nutrients. Ultrasound, as a promising green technology, may influence properties of fibers and proteins and, thus, bioaccessibility. Therefore, this study investigated the effects of high-intensity ultrasound on the characteristics and protein bioaccessibility of protein–fiber suspensions. Suspensions containing different fiber compounds (1 wt.%) and pea protein (5 wt.%) were homogenized using high-intensity ultrasound (amplitude 116 µm, t = 150 s, energy density = 225 kJ/L, $\bar{P}$ = 325 W). Owing to sonication-induced cavitation, the dispersibility of the protein was enhanced, and the viscosity of solutions containing citrus or apple fiber was increased. FE-SEM revealed the formation of different fiber–protein networks during sonication. Even if viscosity is known to have an impact on the bioaccessibility of nutrients, no restrictions on the digestibility of protein were detected during an in vitro digestion. Thus, protein uptake is probably not affected, and ultrasound can be used to modify the technofunctionality of fibers and proteins without any nutritional disadvantages.

## 1. Introduction

Population growth, rising life expectancy, and age-related diseases are future challenges that science must face. Not only because of a human population whose size is predicted to expand to nearly 10 billion people by 2050 [1,2] but also owing to the increasing number of diseases, such as cancer, obesity, and diabetes, it is time to devise ways of eating a healthy and sustainable diet [3,4]. Considering ethical, health, and environmental impacts of conventional meat and animal-product consumption, plant-based nutrition could be a promising alternative. Plant products deliver high amounts of dietary fiber (DF), vitamins, and minerals. Some plants, such as peas, beans, and soy, can act as an excellent source of essential amino acids in daily nutrition, which are fundamental for human metabolism [5,6,7]. Similar to plant proteins, DF also has numerous positive effects on human health. DF are known to reduce the risk of suffering from coronary heart diseases, diabetes, and obesity but, at the same time, are not sufficiently consumed [8,9,10]. The term DF includes poly- and oligosaccharides, lignin, and associated plant substances [11]. In general, DFs are edible plant carbohydrates that are resistant to digestion in the small intestine but are completely or partially fermented in the large intestine. A classification in accordance with their solubility and fermentability is possible. Both properties are closely related to their structure. Irregular structures, found for pectin or β-glucan, promote higher solubilities, mostly by creating higher viscosities, resulting in altered nutrient absorption and leading to fast fermentation. Instead, regular backbones, characteristic for cellulose or hemicellulose, contribute to an insoluble behavior, resulting in increased fecal bulk as well as intestinal transit time and slow fermentation in the colon [12,13,14]. Together, plant proteins, as a more sustainable and healthier alternative to animal protein, and dietary fiber, as a possible health-promoting prebiotic, can be a promising basis for developing future food products. Nevertheless, plant proteins in their native state often exhibit poor functionalities and characteristics, such as low solubility, off flavors, or antinutritive components, resulting in reduced bioavailability [15,16,17,18]. Additionally, owing to industrial extraction and processing, the functionality of pea proteins is further reduced because of denaturation and the formation of aggregates [19,20]. Plant fibers are also known to have poor technofunctional properties, such as low emulsifying or water-binding capacities, and to alter the bioaccessibility of nutrients [21,22,23]. To compensate the poor functional properties of plant protein and improve those of plant fibers, high-intensity ultrasound (US) was used. Ultrasound, as a novel and green technology, is known to induce changes in the structure of proteins and plant fibers [24,25]. The main phenomenon responsible for the efficacy of ultrasound is called cavitation. The essential mechanism of droplet breakup during sonication happens because of acoustic cavitation, which is associated with power dissipation. Sound waves propagate through liquid media, resulting in high-pressure and low-pressure zones. In low-pressure zones, the liquid evaporates, and small vapor-filled bubbles emerge. During alternating pressure cycles, vapor filled bubbles grow and finally collapse, resulting in local high temperatures and pressures as well as in microjets, which release enormous shear forces. The temperature, treatment time, and amplitude are some of the parameters that can be varied during the high-intensity ultrasound treatment of liquid samples because they influence the intensity of the treatment. Higher amplitudes usually result in more effective cavitation. The higher the amplitude, the higher the rate of alternating pressure cycles, and the higher the destructiveness to solid particles [26,27]. As a result, aggregated particles and droplets are ruptured owing to the cleavage of intermolecular hydrophobic interactions, van der Waals forces, and hydrogen bonds [28,29]. There is much literature available on the influence of US on organic substances [29,30,31,32]. However, little is known about the effect of US on the bioaccessibility of plant proteins in the presence of plant fibers. Therefore, the aim of this study was to investigate the influence of plant fibers on in vitro pea protein digestibility in ultrasound-prepared suspensions. 

## 2. Materials and Methods

### 2.1. Materials

Commercial plant fibers (citrus fiber Herbacel AQ Plus Citrus N; citrus fiber Herbacel Classic CF 02; apple fiber Herbacel AQ Plus Apple A 09; oat fiber Herbacel Classic Plus HF 04; and pea fiber Herbacel Classic Plus EF 01) were obtained from Herbafood Ingredients GmbH (Werder, Germany). Pea protein isolate (Empro E 86 HV) was provided by Emsland Stärke GmbH (Emlichheim, Germany). Pepsin from porcine gastric mucosa (504 U/mg, Product number P7000, Lot number BCBZ7879), trypsin (1000–2000 U/mg, Product number T7409, Lot number SLCD9047), and chymotrypsin (40 U/mg, Product number C4129, Lot number SLCH1926) were purchased from Sigma–Aldrich (St. Louis, MO, USA). All the other chemicals used in this study were purchased from either Sigma–Aldrich (St. Louis, MO, USA) or Merck KGaA (Darmstadt, Germany). For SDS–PAGE, 4–20% Criterion™ TGX Stain-Free™ Protein Gel (18 wells, Product number 5678094) for molecular weights between 10 and 250 kDa and Precision Plus Protein^TM^ Standards Unstained marker (Product number 1610363) were obtained from Bio-Rad Laboratories GmbH (Feldkirchen, Germany). Tap water was used for the preparation of the samples; for the preparation of solutions, deionized water was used.

### 2.2. Composition of Plant Dietary Fiber

A general overview of the composition and total dietary fiber content of the plant dietary fibers is given in Table 1. The information was kindly provided by the manufacturer. Soluble (SDF) and insoluble dietary fiber (IDF) contents were determined according to the AOAC 991.43 method and are shown in Table 2. Abbreviations used for different types of fiber are as follows: CF AQ—citrus fiber AQ Plus Citrus N; CF Cl—citrus fiber Herbacel Classic CF 02; AF—apple fiber Herbacel AQ Plus Apple A 09; OF—oat fiber Herbacel Classic Plus HF 04; and PF—pea fiber Herbacel Classic Plus EF 01.

### 2.3. Suspension Properties

#### 2.3.1. Suspension Preparation

Samples containing 5 wt.% pea protein and 1 wt.% plant dietary fiber, acting as a natural stabilizer, were prepared at a volume of 200 mL. The dietary fibers were apple, pea, oat, and two different citrus fibers, varying in their degree of purification. A reference pea protein sample without the addition of plant fiber was also investigated. After mixing the ingredients, the pH of the solution was adjusted to 7, and the solution was stirred for 10 min. For homogenization of the solution, high-intensity ultrasound (UIP 2000; BS2s18 (F), Hielscher Ultrasonics GmbH, Berlin, Germany) was applied under the following conditions in the continuous processing mode: amplitude = 116 µm, t = 150 s, energy density = 225 kJ/L, and $\bar{P}$ = 325 W. The sample was placed in an ice bath and stirred manually during the ultrasound treatment. The temperature of the suspension was determined using a manual thermometer (testo 104, Testo, Lenzkirch, Germany) and did not exceed 40 °C. To determine possible effects of the ultrasound treatment on the pea protein, a sample with untreated pea protein was prepared as well. The energy density was calculated as follows, where P is the power output (W), t is the treatment time (s), and V is the treated volume ($m^{3}$):

(1) $$energy\;density=\frac{P\cdot t}{V}$$

#### 2.3.2. Determination of Particle Size Distribution

The particle size distribution of the untreated and ultrasound-treated pea protein–fiber suspensions was analyzed by applying static laser light scattering using a particle size analyzer LA-950 (Horiba, Retsch Technology GmbH, Haan, Germany). The volumetric particle size distribution was applied to measure the mode and mean values, based on the Fraunhofer diffraction equation. All the suspensions were measured at least in triplicate. 

#### 2.3.3. Rheological Characterization

To measure the rheological characteristics of the suspensions containing 1% fiber and 5% pea protein, the method of Huang et al. [33] with slight modifications was applied. Therefore, a shear rheometer (MCR 102, Anton Paar GmbH, Graz, Austria) equipped with a concentric cylinder setup (CC27/T200/SS, Anton Paar GmbH, Graz, Austria) was used. Samples were homogenized prior to each measurement, avoiding settling of the protein or fiber, and equilibrated at 25 °C for 30 s at a constant pre-shear rate of 0.1 $s^{-1}$. This preliminary section was followed by a controlled shear-rate test consisting of three sections. The first section is characterized by an ascending shear rate ranging from 1 to 100 $s^{-1}$, followed by a constant shear rate of 100 $s^{-1}$ in the second section, and finally ending with a descending shear rate ranging from 100 to 1 $s^{-1}$ in the third section. In each section, 25 measurement points per ramp were recorded to determine the apparent viscosity as a function of the shear rate.

#### 2.3.4. Field-Emission Scanning Electron Microscopy (FE-SEM) of Suspensions

For microstructural analysis, samples were analyzed in a field-emission scanning electron microscope (ZEISS GeminiSEM 500 Nano VP, Carl Zeiss AG, Oberkochen, Germany). An acceleration voltage of 12.00 or 20.00 kV was used, and micrographs at magnifications of ×400 and ×2000 were obtained. Prior to microscopy, samples were freshly prepared, frozen in liquid nitrogen, and subsequently freeze dried. A small amount of each sample was adhered to double-sided conductive tape on an aluminum tracer before the samples were placed in the microscope. 

### 2.4. In Vitro Digestion

#### 2.4.1. Enzyme Solution Preparation

Enzyme solutions for digestion experiments were prepared according to methods used in previous studies [34,35,36]. Therefore, solutions for the simulated gastric phase and intestinal phase were needed. For the simulated gastric phase, pepsin was dissolved in deionized water, obtaining activities around 2520 U/mL in gastric fluid and 63 U/mL in the final sample mixture. For the simulated intestinal phase, enzyme solutions containing trypsin and chymotrypsin were prepared. Both enzymes were dissolved in 1 mM hydrochloric acid (HCL) separately, resulting in activities of 2000 U/mL in the concentrate and 50 U/mL in the final solution for trypsin or 400 U/mL in the concentrate and 10 U/mL in the final sample mixture for chymotrypsin. All the solutions were frozen in aliquots with liquid nitrogen and stored at −20 °C until needed. 

#### 2.4.2. In Vitro Digestion Model

In vitro digestion was performed according to the method used in a previous study [35] with slight modifications, consisting of stomach and intestinal phases. For the stomach phase, 20 mL of the protein–fiber solution (pH 7) and 20 mL of HCL (0.5%) were mixed. After the first sample collection of 2 mL, 1 mL of the enzyme solution containing pepsin was added following incubation for 1 h at 37 °C in a shaking incubator at 250 rpm. Then, another 2 mL of the sample was taken before 40 mL of 150 mM $NaHCO_{3}$ solution (pH 8.5) was added. If the pH did not reach 7, it was adjusted using 1 M NaOH solution. Before adding 1 mL of the enzyme solutions containing trypsin and chymotrypsin, another 2 mL of the sample was taken for further analysis. The solution was incubated during the intestinal phase for 2 h at 37 °C in a shaking incubator (250 rpm). Samples (2 mL) were taken every 15 min during the first hour and every 30 min during the second hour. All the samples were inactivated in a hot water bath for 15 min following storage on ice. After the whole in vitro digestion was finished, every sample was centrifuged for 15 min at 12,000 rpm. The clear supernatants were collected and frozen until the analysis. A control containing only digestive enzymes in deionized water was included in the assay for measuring the free amino groups of the pure digested enzymes. The increase in free amino groups was assumed as an indicator for the enzymatic degradation of the protein during the in vitro digestion (see Section 2.4.3). 

#### 2.4.3. Determination of Free Amino Groups—OPA Method

To follow the progress of the protein digestion, the amount of free α-amino groups was determined according to the OPA spectrophotometric assay described elsewhere [37]. Free amino groups form complexes with ortho-phthaldialdehyde (OPA) in the presence of SDS and mercaptoethanol in an alkaline milieu, which are detectable at a wavelength of 340 nm. To prepare the working solution, 40 mg of OPA was dissolved in 1 mL of methanol, mixed with 25 mL of sodium tetraborate solution (100 mM), 10 mL of SDS solution (5 wt.%), and 100 μL of mercaptoethanol and filled to a volume of 50 mL with deionized water. The reagent must be prepared freshly every day of the analysis and stored in darkness. For the analysis, 100 μL of the sample and 1000 μL of the reagent were mixed, incubated for 6 min in the dark, and finally photometrically measured at a wavelength of 340 nm (Lambda 365 UV–VIS Spectrophotometer, Perkin Elmer, Waltham, MA, USA). For dilution of the samples and as a blank, deionized water was used. For the calibration curve, cysteine hydrochloride monohydrate in different concentrations between 0.1 and 5 mM was measured. Free α-amino groups referred to the protein content in the sample and were determined using the Biuret assay (see Section 2.4.5).

#### 2.4.4. Sodium Dodecyl Sulfate Polyacrylamide Gel Electrophoresis (SDS–PAGE)

To gain more information about the molecular weight and structure of the native, ultrasound-treated, and digested protein, SDS–PAGE was performed. Native pea protein samples were investigated directly after the ultrasound treatment and after digestion. The gel was prepared and filled following the instructions of the manufacturer, and the appropriate marker was utilized. A protein content of 100 μg in a 10 μL sample per slot was necessary. The protein content was determined beforehand using a Biuret assay (see Section 2.4.5). After running the SDS–PAGE, a Gel Doc EZ system, including Image Lab software (Version 5.2 build 14, Bio-Rad Laboratories GmbH, Feldkirchen, Germany) and a Gel Doc EZ Stain-Free Sample Tray (Bio-Rad Laboratories GmbH, Feldkirchen, Germany), was utilized for the analysis. 

#### 2.4.5. Biuret Assay

For quantifying untreated and treated pea protein solutions, the Biuret assay based on the work of [38] was utilized. Therefore, a Biuret reagent containing 9.4 mM copper sulfate, 28.56 mM potassium sodium tartrate, and 831 mM sodium hydroxide was prepared. For the assay, 800 μL of the Biuret reagent and 200 μL of the diluted sample were mixed. Complexation was reached during 20 min of incubation at 37 °C in a shaking incubator (250 rpm). Afterward, the absorption was measured at a wavelength of 540 nm (Lambda 365 UV-VIS Spectrophotometer, Perkin Elmer, Waltham, MA, USA). Deionized water was used for dilution and as a blank. Bovine serum albumin was utilized for establishing a calibration curve in the concentration range 0.04–5 g/L. 

### 2.5. Statistical Analysis

All the experiments were performed at least in duplicate, unless stated otherwise. Standard deviations and averages were calculated from these measurements. Student’s *t*-tests were performed to evaluate the difference between the samples. Statistical significance was considered when *p* < 0.05. Statistical analysis was performed using Microsoft Excel (Version 2307).

## 3. Results and Discussion

### 3.1. Influence of High-Intensity Ultrasound on Pea Protein

**Figure 1:**
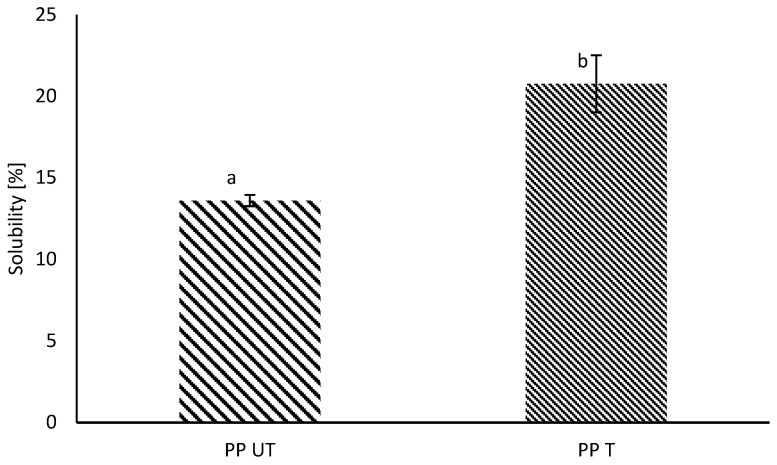
Water solubility (%) of untreated (UT) and sonicated (T) pea protein (PP). Different letters indicate significant differences (*p* < 0.05).

In general, commercial pea protein isolates exhibit poor solubility, which is the major concern for their application in food products. Poor solubility is often a consequence of different extraction conditions during protein fractionation, such as high temperatures or isoelectric precipitation [39]. Physical and thermal stresses, such as shear forces during mixing or spray drying, may influence the secondary and tertiary structure of proteins, leading to partial or complete denaturation. Particles may form large multimeric protein particles or protein–nonprotein aggregates, which have poor solubility. Nonprotein substances, such as insoluble dietary fiber, can act as nucleation sites, inducing the formation of insoluble aggregates [40]. Therefore, a low solubility for the investigated PP was expected. After ultrasound treatment, a slight increase of around 7% was observed, which is illustrated in Figure 1. 

The increase in the solubility of the pure pea protein solutions agrees with the results of previous studies, where increases in the solubility of different plant proteins after US-treatment were observed [28,41,42,43]. In general, it is assumed that sonication has no influence on the primary structure of proteins, which is in line with the results from SDS–PAGE. Rather, US leads to changes in secondary and tertiary structures [44,45]. Owing to ultrasound-induced cavitation, noncovalent bonds, such as hydrogen bonds, or hydrophobic interactions are disrupted, resulting in the unfolding of the protein molecule [39]. The higher solubility is attributable to the exposure of hydrophilic regions owing to unfolding as well as the formation of soluble protein aggregates [46]. Hence, interactions between water and protein molecules and, therefore, solubility are enhanced. The newest findings also suppose the formation of soluble complexes between pea protein and indigenous dietary fiber, which increases the solubility [40]. 

**Figure 2:**
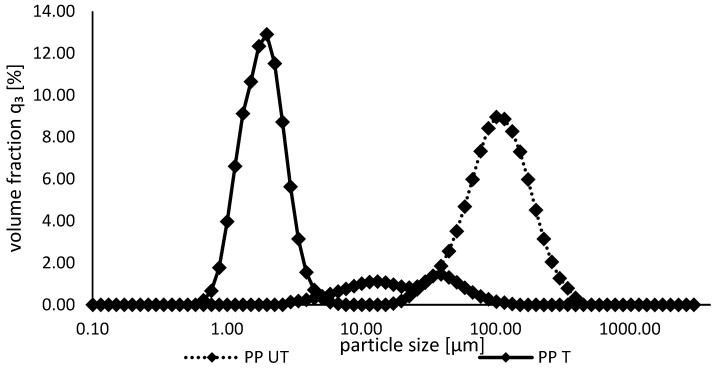
Particle size distributions of untreated und ultrasound-treated pea protein suspensions.

With regard to solubility, particle size can be an influencing factor. In general, smaller protein aggregates are more soluble. They present a larger surface area with more functional groups, which can be in close contact with water molecules [39,46]. As shown in Figure 2, the particle size of the pea protein isolate was significantly reduced after the ultrasound treatment, which is in line with observations of other studies [39]. The curves switched from a broad monomodal distribution, with a mode value of 99.5 µm, to a narrower particle size distribution with an increased volume fraction and a decreased mode value of 1.85 µm (Ultrasound-induced cavitation leads to the disruption of protein aggregates by disrupting intermolecular interactions, resulting in smaller particles [29]. Additionally, sonication leads to the partial disruption of hydrogen bonds and hydrophobic bonds, resulting in the unfolding and swelling of protein macromolecular chains, which, in turn, leads to even more disintegration [47].

**Figure 3:**
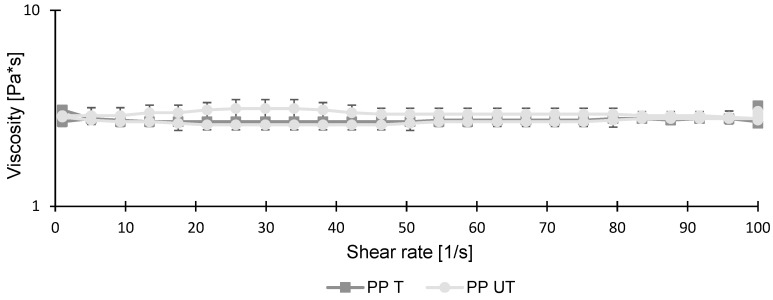
Viscosities of untreated (UT) and treated (T) pea protein (PP) suspensions (10 wt.%) as a function of shear rate ranging from 0 to 100 $s^{-1}$.

In Figure 3, the viscosities of the untreated and treated pea protein solutions are shown. They indicate a nearly ideal Newtonian fluid behavior. Although ultrasonication led to a significant reduction in particle size (Figure 2), it had no influence on viscosity. As already discussed in previous studies, smaller particle sizes usually result in reduced viscosities [24,48]. However, because the initial viscosity of the untreated sample was already low, the particle size reduction had no further effect. Similar results were obtained when treating whey protein isolate with high-intensity ultrasound. A significant reduction in particle size, from 287.75 nm to 190.60 nm, was observed when increasing the treatment time and amplitude. For viscosity measurements, no changes were investigated [49].

**Figure 4:**
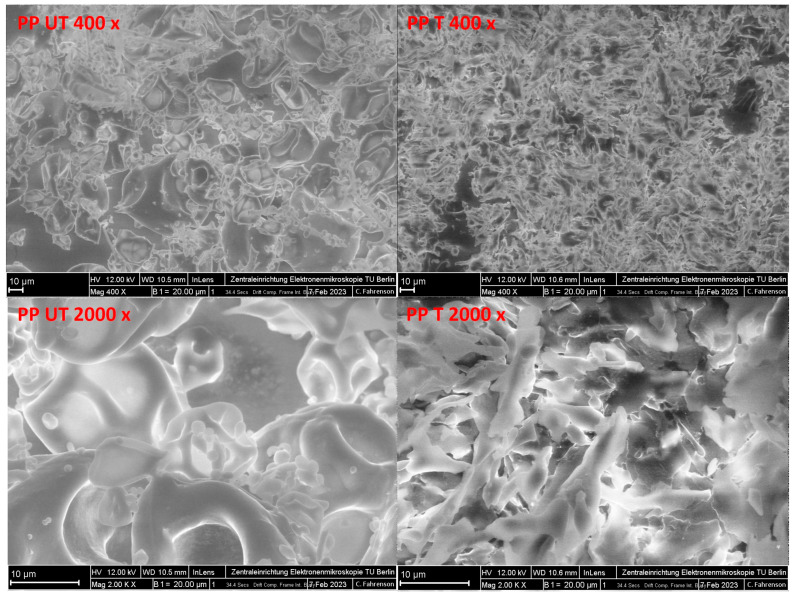
FE-SEM images (400× and 2000× magnifications, scale bar: 10 µm) of untreated (UT) and ultrasound-treated (T) pea protein samples (PP).

The FE-SEM images of the untreated and ultrasound-treated pea proteins are illustrated in Figure 4. For the untreated pea protein, large cubic particles consisting of ovoid and spherical shapes with wrinkles and indentations are clearly identified. The images are similar to those obtained by other authors [50]. Attached to the surface of the protein particles, individual globular entities and aggregates are observed, as well as some elongated particles, probably representing mineral and fiber residues, as reported elsewhere [51]. After sonication, totally different structures are observed. The initial homogenous structures are changed to inhomogeneous, flat, and flaky particles consisting of different sizes and shapes. Recently, similar flake-like and rod-shaped structures in water-soluble pea protein isolate were reported by Gao et al. [40] and for soy protein by Zou et al. [47]. They speculated that the deformation of globular structures to a stretched and flatter conformation of soy protein molecules was due to shear forces. Furthermore, the subsequent reassociation of primary aggregates and formation of soluble subassociates of the pea protein are suspected. In a study investigating the effect of ultrasound on soy protein isolate, large and heterogenous structures were also observed, which suggested that ultrasonic treatment leads to the unfolding of protein molecules and, therefore, increases the exposure of SH groups and hydrophobic groups at the surface. During freeze-drying, these functional groups could interact with each other, forming larger aggregated structures consisting of irregular sizes [52]. However, as revealed by SDS–PAGE, no aggregation was observed, indicating that aggregates are noncovalently bonded and might be formed during freeze-drying. This might be the reason for the significantly smaller particle sizes measured during static light scattering (Figure 2). 

### 3.2. Influence of High-Intensity Ultrasound on Suspensions Containing Plant Fiber and Pea Protein

**Figure 5:**
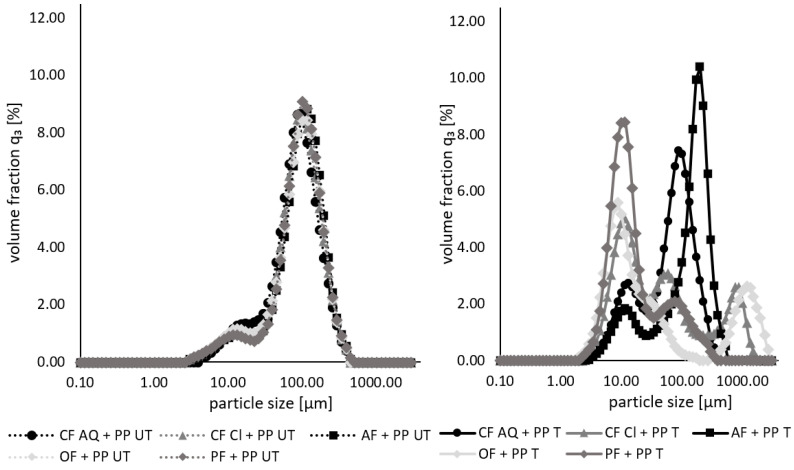
Particle size distributions of untreated (UT, **left**) and ultrasound-treated (T, **right**) pea-protein and fiber suspensions. (PP—pea protein; CF—citrus fiber; AF—apple fiber; OF—oat fiber; PF—pea fiber).

The particle size measurements of the untreated and treated suspensions containing both the different plant fibers and pea protein are presented in Figure 5. For untreated samples, no differences in particle size are observed. It is obvious that the particle size distribution is nearly similar to that of native pea protein in Figure 2. This phenomenon might be due to the formation of aggregates or complexes during the mixing of both components. This assumption is supported by findings of another study. When investigating the effect of wheat-bran dietary fiber on the gluten structure under different thermal processing conditions, interactions between the fiber and protein were observed. Even at room temperature, fiber particles wrapped around protein molecules because of strong hydrogen bonds [53]. Yet, the particle size distribution for the sonicated samples looks different. From a nearly monomodal distribution, ultrasonication leads to a shift to a bi- or unimodal distribution. A similarity of all the curves is a peak at around 10 µm for various volume fractions, possibly representing disrupted pea protein. For CF AQ- and AF-enriched pea protein solutions, the second peak is similar to that of the respective sonicated fiber [54]. For the other three remaining fiber compounds, the second (or in the case of CF Cl, the second and third) peak(s) is/are at a random size. It can be speculated that the covering of plant fiber particles around protein aggregates acted similar to a shield, protecting protein aggregates from disruption. Wen et al. [53] observed a swelling of the fiber compounds while wrapped around the protein, becoming tighter during heating. This swollen fiber covering protected the gluten from heat, whereby it remained unaffected. This protective effect might be transferable to the results of the current study. The protection of the fiber-surrounded protein against shear forces induced by cavitation is conceivable. Only when the attached fiber compounds were removed, a particle size reduction of the pea protein took place. An additional assumption can be the disruption of pea-protein aggregates owing to collisions between the protein and dietary-fiber particles. This explains the irregular particle size distribution of all the samples after the ultrasound treatment. There were fiber fragments detected as well as partially disrupted pea-protein particles.

**Figure 6:**
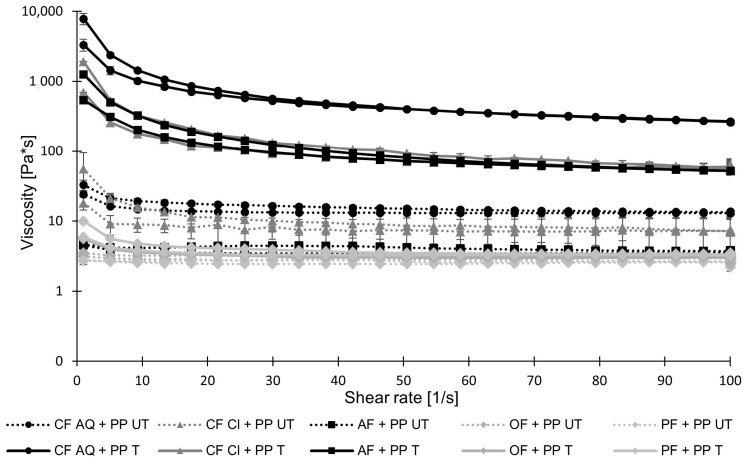
Viscosities of untreated (UT) and treated (T) pea-protein and fiber suspensions, containing 10 wt.% pea protein and 1 wt.% dietary fiber suspended in water, as a function of shear rate ranging from 0 to 100 $s^{-1}$ (PP—pea protein; CF—citrus fiber; AF—apple fiber; OF—oat fiber; PF—pea fiber).

Flow curves of untreated and treated suspensions containing pea protein and plant fiber compounds are shown in Figure 6. After the sonication of CF AQ + PP and AF+PP, an increase in viscosity is observed, as already observed for pure sonicated fiber suspensions [54]. It is assumed that the unraveled microstructure of sonicated fiber particles in a previous study [54] is dominant and undisturbed by pea-protein suspensions. Therefore, water can be held in the structure and finally result in increased viscosities. Interestingly, for sonicated CF Cl + PP solutions, an increase in viscosity occurred as well. The previously investigated pure CF Cl solution exhibited no increase in viscosity after ultrasonication treatment, which was attributed to an insufficiently formed pectin network. Vercet et al. [55] described pectin molecules as the main agent for physical interactions. The incorporation of smaller particles in the pectin network is thought to be beneficial for increasing viscosity. Viscosity usually increases when particles in the solution are in close contact with each other, which increases friction and, therefore, flow resistance [56]. For CF Cl and pea protein, in this study, small protein molecules might act as a filler in the pectin network, finally causing an increase in flow resistance after sonication. For OF and PF, after the ultrasound treatment, no increase in viscosity is observed, which agrees with previous measurements of pure fiber suspensions [54]. There is no viscosity-increasing effect between the protein and either the oat or pea fiber. 

**Figure 7:**
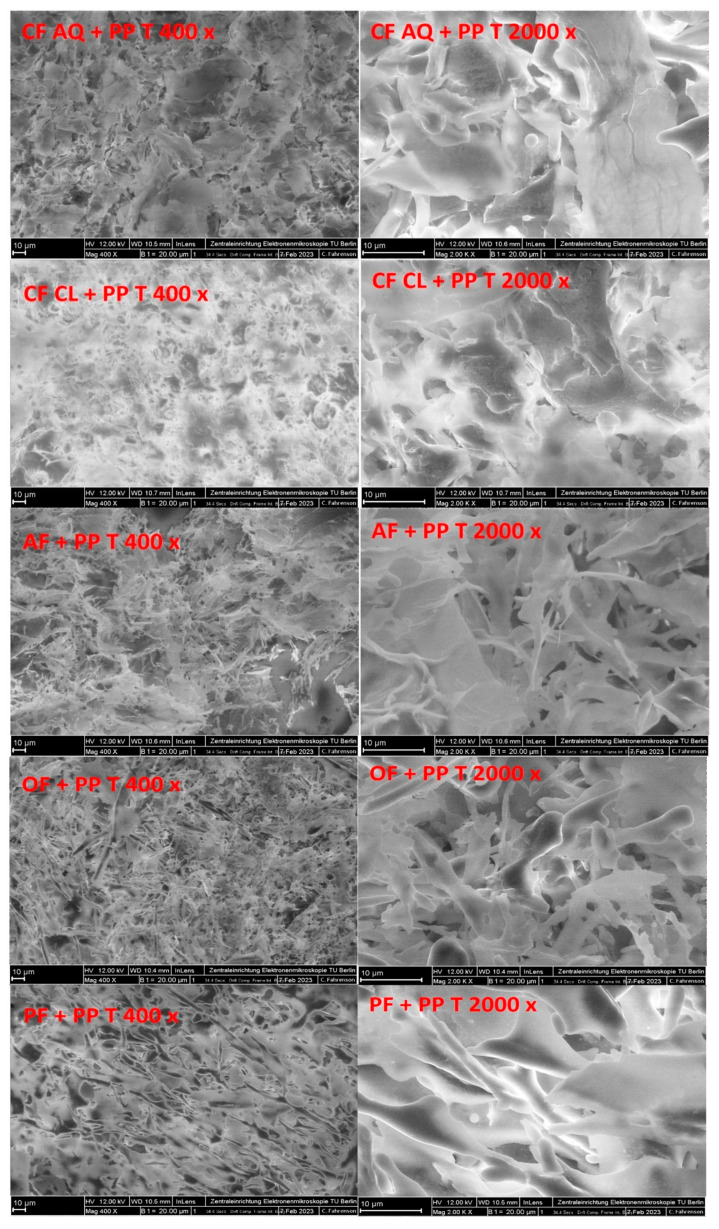
SEM images (400× and 2000× magnifications, scale bar 10 µm) of untreated (UT) and ultrasound-treated (T) pea protein and fiber samples. (PP—pea protein; CF AQ—citrus fiber AQ; CF Cl—citrus fiber Cl; AF—apple fiber; OF—oat fiber; PF—pea fiber).

In Figure 7, the FE-SEM images of all the treated suspensions containing plant fiber and pea protein are shown. Initially, a protein network, similar to that generated in pure pea-protein suspensions (Figure 4), is formed. However, for OF- and PF-enriched protein suspensions, individual fiber fragments incorporated in the network are clearly detectable. As identified in a previous study [54], for CF AQ- and AF-fortified solutions, thin layered structures are observed again. Therefore, the fiber fragments obtained after sonication are embedded in the protein network and formed a more-or-less homogenous joint network. In another study investigating dietary fiber–gluten interactions, fibers tended to spread in the gluten network and interact with each other. The entanglement or self-aggregation of high-molecular-weight SDF can be inhibitory for intermolecular interactions of the protein, resulting in an interrupted network formation [57]. Additionally, increased particle sizes can be a promoting factor for disturbed protein-network formation [58]. For OF and PF, the network seemed to be more homogenous whereas for CF AQ and AF, a more fragmented appearance is observed. This could be an indicator of a truly disturbed network owing to higher amounts of SDF in both compounds or could be attributed to single fiber layers, as already observed for pure fiber solutions [54]. Further work is needed to differentiate these two options. For CF Cl and pea protein, a cloudy and dense protein-fiber network is observed. The previous hypothesis of increased viscosity owing to the incorporation of ruptured pea protein particles in the fiber network might be applicable. The impression is given that the fiber network of CF Cl revealed in a study by Kalla–Bertholdt et al. [54] is still present but filled with protein particles. Furthermore, the resulting networks of all the samples after sonication imply a more compact and denser structure. Owing to the unfolding of the protein, the exposure of functional groups, such as SH groups and hydrophobic groups, increases at the surface [52]. Therefore, the number of interactions between the fiber and protein also increases owing to electrostatic interactions, such as hydrogen bonding or hydrophobic and van der Waals forces [59]. In another study investigating properties of pectin-pea protein-isolate mixtures, “particle pressing” was observed. The authors concluded that when the amino group of the protein interacts with the carboxylic group of the pectin, particles are pressed, resulting in smaller particles [50]. Possibly, this phenomenon also occurred for the fiber samples investigated in this study, which presented more compact structures. In summary, after sonication, a protein network was formed for all the investigated fiber samples. Owing to sonication, the unfolding of the protein and rupture of the fiber particles led to more functional groups being exposed. Therefore, interactions between the fiber and protein resulted in a more-or-less dense structure.

### 3.3. In Vitro Digestion Model

**Figure 8:**
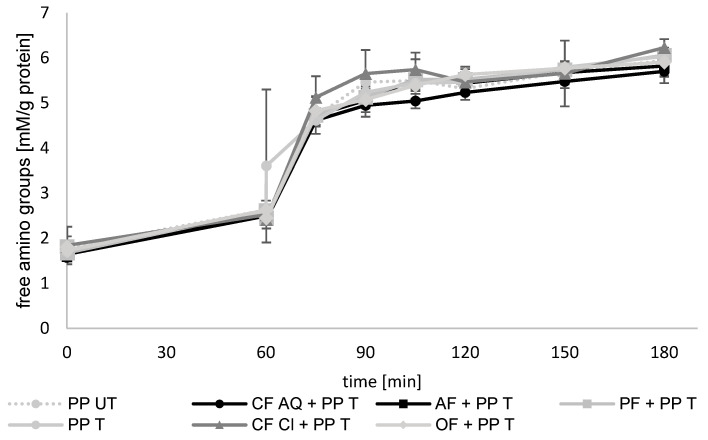
Free amino-acid groups (mM/g protein) in untreated (UT) and treated (T) pea-protein and fiber-enriched suspensions. Gastric phase was between 0 and 60 min and intestinal phase followed between 60 and 180 min. (PP—pea protein; CF AQ—citrus fiber AQ; CF Cl—citrus fiber Cl; AF—apple fiber; OF—oat fiber; PF—pea fiber).

**Figure 9:**
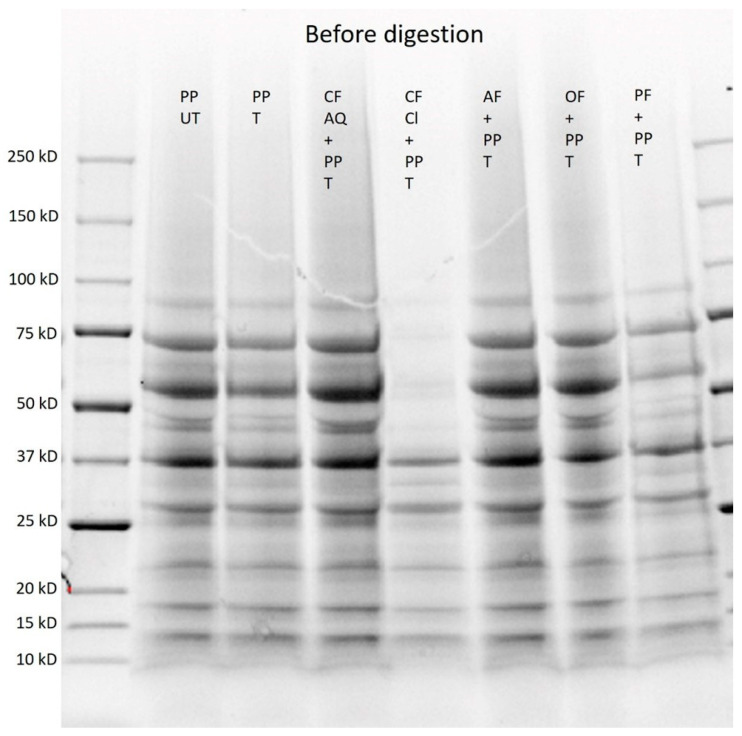
SDS–PAGE of untreated (UT) and treated (T) pea-protein and fiber-enriched protein solutions before digestion. (PP—pea protein; CF AQ—citrus fiber AQ; CF Cl—citrus fiber Cl; AF—apple fiber; OF—oat fiber; PF—pea fiber).

To gain more information about the influence of the dietary fiber and ultrasound treatment on the bioaccessibility of pea protein, an in vitro digestion was conducted. As an indicator for protein hydrolysis during enzymatic degradation, free accessible amino groups were measured as millimoles of cysteine hydrochloride monohydrate equivalents per initial protein (Figure 8). The gastric phase had a duration of 60 min, and the intestinal phase was conducted for a further 120 min, directly after the gastric phase. Therefore, the intestinal phase started at 60 min and ended after 180 min, as shown in Figure 9.

The digestibility of pea proteins after several technological treatments has been investigated in many studies [35,60,61,62]. The results vary, including increased, decreased, or unaltered hydrolysis. Several explanations for these findings have been discussed. The protein source, size, and conformation as well as degree of denaturation or formation of aggregates are thought to be determinants for protein digestion [7,63]. In addition, the purity of compounds, such as flours, concentrates, or isolates, are mentioned as influencing factors because of possible remaining antinutritive components, such as trypsin inhibitors or tannins [64]. Another factor that is crucial for protein bioaccessibility can be the presence of dietary fiber. Highly viscous fibers, such as pectin, can alter the viscosity of the digesta, resulting in decreased digestion of protein [65]. By evaluating the results of the present study, no differences were found between untreated and ultrasound-treated proteins or between sonicated samples containing plant dietary fiber. During the gastric phase, a slight increase in free amino groups was detected. During the first 15 min of the intestinal phase, most of the breakdown of proteins occurred in accordance with results reported elsewhere [66]. This was attributed to the addition of trypsin and chymotrypsin, which have higher activities compared to pepsin. Pepsin degrades larger peptides and trypsin as well as chymotrypsin, generating low-molecular-weight peptides and resulting in a rapid increase in free amino groups. Regarding the plant fiber in this study, the revealed structural and functional properties, especially the network formation (Figure 7) or increases in viscosity (Figure 6), had no remarkable effect on the breakdown of protein and, therefore, no effect on the bioaccessibility.

By summarizing the findings of the in vitro digestion for insights into the bioaccessibility of the protein, it can be stated that for the pea protein used in this study and for the applied ultrasound treatment and fiber enrichment, no differences were observed. In general, the results of in vitro studies should be taken with caution because the bioaccessibility for every protein source can be totally different, and no generalization can be made [67]. Additionally, the results must be corroborated with in vivo studies where all aspects and dynamics of digestion are considered [68]. 

To gain more insights into the digestion products, an SDS–PAGE was run. Therefore, untreated and ultrasound-treated pea protein was investigated before digestion. Additionally, pea-protein solutions containing different plant fibers, were investigated before digestion (Figure 9). 

For the untreated and sonicated samples, no differences in molecular weight were observed, which is in accordance with the results of Xiong et al. [44] for pea-protein isolate. This was expected because ultrasound is proposed to alter secondary and tertiary structures but not the primary structure [41]. Furthermore, the detected molecular weights correspond with the typical SDS–PAGE profile of individual subunits for pea protein. The most dominant subunits are convicilin at around 70 kDa, legumin at around 60 kDa, and vicilin at around 50, 31–37 kDa, and 10–12 kDa. Vicilin is a trimeric protein composed of 50 kDa and 30 kDa subunits and minor components that have molecular weights less than 19 kDa. Furthermore, bands at 37 kDa can be described as legumin A and at 17 kDa as legumin B [69,70]. Interestingly, for suspensions containing CF Cl, the bands are much weaker, and the bands for legumin at 60 kDa and for convicilin at 70 kDa disappeared. This might be attributed to polyphenolic components in the citrus-fiber compound. Polyphenols are known to bind to proteins and, therefore, can induce conformational changes [71,72]. As previously investigated for whey-protein isolate, polyphenols can form conjugates with proteins, increasing their molecular weight and, therefore, slowing their migration [72]. Yet, the polyphenol content in the CF Cl compound was comparatively low and, therefore, further work is needed to examine this phenomenon. 

**Figure 10:**
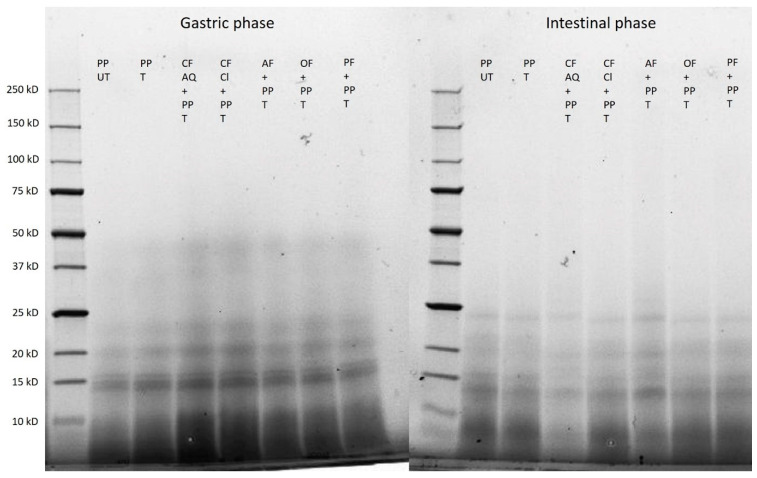
SDS–PAGE of untreated (UT) and treated (T) pea-protein and fiber-enriched protein solutions after gastric and intestinal digestions. (PP—pea protein; CF AQ—citrus fiber AQ; CF Cl—citrus fiber Cl; AF—apple fiber; OF—oat fiber; PF—pea fiber).

The electrophoresis patterns after gastric and intestinal digestions are shown in Figure 10. Clearly, no differences between the samples are detectable. This demonstrated that the breakdown of the protein proceeds during the hydrolysis of pepsin, trypsin, and chymotrypsin. The molecular weight is constantly decreasing. Whereas, after the gastric phase, molecular weights between 50 kDa and 15 kDa are still detectable, after the later intestinal phase, only faint bands between 25 kDa and 15 kDa are found, and the majority is smaller than 10 kDa. This led to the assumption that no intact residual protein is left. These findings agree with results of other studies [62]. Furthermore, it can be concluded that neither dietary fiber enrichment nor ultrasonication led to modifications or restrictions in bioaccessibility of protein and finally resulted in complete hydrolyzation.

## 4. Conclusions

The study of functional and structural changes in pea-protein and protein–fiber suspensions induced by ultrasound led to a better understanding of interactions between both components. The hypothesized effect of dietary fiber and ultrasound application did not alter the bioaccessibility of the pea protein. Nevertheless, an increase in viscosity for apple- and citrus-fiber enriched protein solutions was observed. This was attributed to the higher SDF content and initial small particle sizes in the fiber compounds as well as the increased water-binding capacity of AF and CF AQ after the ultrasound treatment. Additionally, a similar network formation for these two fiber compounds was observed, which seemed to be beneficial for water incorporation. However, for CF-CL-enriched protein suspensions, an increase in viscosity was observed, which was not detectable for pure CF-Cl solutions. This was attributed to the incorporation of small protein particles in the network formed by the fiber. Therefore, it can be concluded that not only the pectin content but also the particle size and type, processing history, and purity of the commercial fiber compound are determining factors.

For particle size measurements of the pea protein, the results were as expected. The hypothesized reduction in particle size, owing to cavitation-induced shear forces, was proven. This was achieved owing to the disruption of protein aggregates, which simultaneously had a positive impact on the solubility. Sonication led to the unfolding of molecules, which enhanced interactions between the water and protein. For particle size measurements in fiber-enriched protein suspensions, irregular distributions were observed. This was attributed to the protective effect of fiber particles, which were wrapped around protein molecules. For the investigations of the bioaccessibility of pea protein in the presence of plant fiber and ultrasound treatment, no differences were found. Furthermore, SDS–PAGE indicated complete hydrolysis. In general, these results should not be overestimated because in vitro models are simplified compared to in vivo conditions. Nevertheless, these results are promising for further investigations of the incorporation of plant fiber in protein-enriched foods and the application of high-intensity ultrasound in the food industry.

## Figures and Tables

**Table 1:** Fat, carbohydrate, protein, total dietary fiber, and salt contents of the plant dietary fibers.

	Fat Content (%)	Carbohydrate Content (%)	Protein Content (%)	Salt Content (%)	Total Dietary Fiber Content (%)
CF AQ	≤1	≤1	5	1.3	90
CF Cl	<1	6	6	0.4	64–82
AF	≤1	≤1	9	1	85
OF	≤1	≤1	≤1	0.1	Min. 90
PF	1	7	7	0.03	70–90

**Table 2:** Insoluble and soluble dietary fiber contents (% DM) of the plant dietary fibers.

	Insoluble Dietary Fiber (%)	Soluble Dietary Fiber (%)
CF AQ	74.56	14.57
CF Cl	39.9	23.3
AF	71.91	11.14
OF	94.27	0.21
PF	72.91	4.39

## Data Availability

The datasets generated for this study are available on request to the corresponding author.
